# Hydrogel based protein biochip for parallel detection of biomarkers for diagnosis of a Systemic Inflammatory Response Syndrome (SIRS) in human serum

**DOI:** 10.1371/journal.pone.0225525

**Published:** 2019-12-02

**Authors:** Anne Stumpf, Thomas Brandstetter, Johannes Hübner, Jürgen Rühe

**Affiliations:** 1 Laboratory for Chemistry and Physics of Interfaces, Department of Microsystems Engineering, University of Freiburg, Georges-Koehler-Allee, Freiburg, Germany; 2 Division of Pediatric Infectious Diseases, Dr. von Hauner Children's Hospital, Ludwig Maximilian's University, Munich, Germany, Lindwurmstr, Munich, Germany; Chung-Ang University College of Engineering, REPUBLIC OF KOREA

## Abstract

The Systemic Inflammatory Response Syndrome (SIRS), a sepsis related inflammatory state, is a self-defense mechanism against specific and nonspecific stimuli. The six most extensively studied inflammatory biomarkers for the clinical diagnosis of SIRS are interleukin 4 (hIL-4), interleukin 6 (hIL-6), interleukin 10 (hIL-10), tumor necrosis factor alpha (hTNF-α), interferon gamma (hIFN-γ) and procalcitonin (hPCT). These biomarkers are naturally present (but usually only at low concentration) in SIRS infected patients [1, 2] and thus the development of a highly sensitive detection method is of major clinical interest. However, the existing analytical techniques are lacking in required analytical sensitivity and parallel determination of these biomarkers. We developed a fast, easy and cost-efficient protein microarray biochip where the capture molecules are attached on hydrogel spots, enabling SIRS diagnosis by parallel detection of these six clinically relevant biomarkers with a sample volume of 25 μl. With our hydrogel based protein microarray biochip we achieved a limit of detection for hIL-4 of 75.2 pg/ml, for hIL-6 of 45.1 pg/ml, for hIL-10 of 71.5 pg/ml, for hTNF-α of 56.7 pg/ml, for IFN-γ of 46.4 pg/ml and for hPCT of 1.1 ng/ml in spiked human serum demonstrating sufficient sensitivity for clinical usage. Additionally, we demonstrated successful detection of two relevant SIRS biomarkers in clinical patient samples with a turnaround time of the complete analysis from sample-to-answer in less than 200 minutes.

## Introduction

A major diagnostic challenge for rapid detection is the parallel detection of different biomarkers at the same time and in the same sample. Existing diagnostics, e.g. enzyme-linked immunosorbent assays (ELISA) are incapable to fulfill these requirements, because the detection is limited to only one biomarker per ELISA test. For six biomarkers, for example, six samples, respectively six ELISAs are required for the detection of six biomarkers resulting in a time-, sample-, and cost-consuming detection method [1]. This exemplified the urgent need of technologies for the fast and parallel detection of different biomarkers in low sample volume formats making diagnostic results available within short time that will greatly improve the detection and monitoring of disease and guides patient therapy.

Highly sensitive tests are also urgently needed for the diagnosis of disease with low abundant biomarkers and for patients with limited amount of blood (e.g. neonates and premature babies) [2]. Trying to achieve such sensitivities, signal amplification methods like immune PCR are applied. However, these methods require additional steps like, in case of the immune PCR, the PCR thermocycling subsequent to the immune reaction and thus increase the complexity of the detection systems. Furthermore, additional reagents are required making the detection system substantially more expensive.

To overcome these obstacles, such as parallel detection and sufficient sensitivity, a microarray is a widely employed format for high-throughput multiplex analysis of biomolecules, such as DNA [3–5] and proteins [6]. As reported, protein microarrays were developed for a variety of diagnostic applications providing sufficient sensitivity and the possibilities for miniaturization and parallelization [5]. For protein microarrays, the molecules are usually immobilized via covalent, physical or affinity based binding [7]. Therefore, the most common fabrication method for protein microarrays are based on substrate materials with surface modifications [8] implemented by e.g. amine or succinimidyl ester chemistry [9]. Major issues of these techniques are the complex and time consuming fabrication process resulting in high costs. To overcome the complex and time consuming fabrication process, hydrogel based platforms are a prospective way for immobilization of the biomolecules. As reported, hydrogel based platforms are used for different applications in the field of diagnostics [10,11].

In this work, we demonstrate an easy and fast one-step fabrication of the hydrogel based protein microarray biochip providing a cost-efficient platform for diagnostic tools [10]. The one-step fabrication method enables simultaneous attachment of copolymer and proteins onto the substrate and furthermore no surface activations and modifications are required enabling a fast fabrication. The hydrogel creates a protective hydrate shell surrounding the proteins increasing their durability. Additionally, the one-step hydrogel based protein microarray fabrication provides a 3D matrix enabling a high density of the immobilized capture antibodies [12–14].

Detection of SIRS was chosen as diagnostic application for the hydrogel based protein microarray biochip; SIRS is a nonspecific disease state caused by inflammation, trauma, infection, ischemia or a combination of these and is also often a precursor to sepsis, severe sepsis and septic shock [15]. The prevalence of SIRS is high, affecting approximately one-third of all in-hospital patients [16] with an associated mortality rate of approximately 7% within 28 days [16]. In the event that SIRS evolves to sepsis (approx. 26% of SIRS infected patients), a severe sepsis (approx. 18% of SIRS infected patients) or a septic shock (approx. 4% of SIRS infected patients) with 28-day mortality rates of 16% (for sepsis) and 20% (for severe sepsis) and 46% (for septic shock) is expected [17]. The biomarkers hIL-4, hIL-6, hIL-10, hTNF-α and hIFN-γ are cytokines and are of crucial importance for the immunological and inflammatory reactions associated with sepsis [17]. The Food and Drug Administration (FDA) has approved PCT as inflammatory biomarker for the diagnosis of sepsis, respectively for SIRS as a precursor of sepsis. The “gold standard” for the analysis of the six SIRS relevant biomarkers is currently ELISA. Trying to overcome the aforementioned limitations of the ELISA method, microarrays for the parallel detection of laboratory biomarkers were published [18,19]but all these microarrays were developed for the diagnosis of sepsis, and with hIL-6 and hPCT only two biomarkers, also related for SIRS detection, were applied in these test. As the SIRS biomarkers are of low abundance in human serum of infected patients, a high sensitivity of the diagnostic tool is required.

To circumvent these limitations, we report here a fast, easy and cost-efficient process for protein microarray generation where the antibodies are immobilized on hydrogel spotted onto an unmodified standard plastic substrate. This protein microarray enables the parallel detection of the six SIRS relevant biomarkers by sandwich immunoassay where the amount of biomarkers is detected via Cy5-labeled streptavidin in a fluorescence reader. To demonstrate applicability of the sandwich immunoassay and the hydrogel based protein microarray, six relevant biomarkers for the diagnosis of SIRS are studied. As sample material for the validation, spiked human serum as well as clinical patient samples are used.

## Materials and methods

### Materials

#### Substrate

Unmodified injection molded cyclo-olefin polymers (COP) substrates (microfluidic ChipShop GmbH, Jena, Germany) comprising a 36 mm long channel with a width of 3000 μm and a depth of 200 μm and a subsequently connected 75 μl collection chamber was used. As the microarray is immobilized in the channel and the channel volume is 21.6 μl, the sample volume is set to 25 μl ensuring complete filling of the channel. The substrates were initially cleaned with 50% (v/v) 2-Propanol (99.8% p.a., Carl Roth GmbH + Co. KG, Germany) and deionized (DI) water in a ultrasonic bath (Sonorex Super RK 100 H, Bandelinelectronic GmbH & Co. KG, German) for 5 minutes to remove impurities and residues, and was subsequently dried with a nitrogen jet and stored at 4°C in a dark place until usage.

#### Proteins

For the applied sandwich immunoassay, specific capture and detection antibodies (Table 1) against the six biomarkers as well as recombinant human proteins for the tests with spiked buffer and human serum matrices were used.

**Table 1:** Overview of the applied antibodies and proteins.

**Capture antibodies**	**Order number**	**Supplier**
Human IL-4 Antibody	MAB604	R&D Systems
Human IL6 Antibody	MAB206	R&D Systems
Human IL-10 Antibody	MAB2172	R&D Systems
Human TNF-α Antibody	MAB610	R&D Systems
Human IFN-γ Antibody	MAB2852	R&D Systems
Human calcitonin	4C10	HyTest
**Detection antibodies**	**Order number**	**Supplier**
Human IL-4 Biotinylated Antibody	BAF204	R&D Systems
Human IL-6 Biotinylated Antibody	BAF206	R&D Systems
Human IL-10 Biotinylated Antibody	BAF217	R&D Systems
Human TNF-α Biotinylated Antibody	BAF210	R&D Systems
Human IFN-γ Biotinylated Antibody	BAF285	R&D Systems
Human Procalcitonin Biotinylated Antibody	4PC47	HyTest
**Proteins**	**Order number**	**Supplier**
Recombinant Human IL-4	204-IL	R&D Systems
Recombinant Human IL-6	206-IL	R&D Systems
Recombinant Human IL-10	1064-IL	R&D Systems
Recombinant Human TNF-α	210-TA	R&D Systems
Recombinant Human IFN-γ	285-IF	R&D Systems
Recombinant Human Procalcitonin	8PC5	HyTest

Cy5-streptavidin (conjugate, VWR International GmbH, Germany) was used for labeling of the biotinylated detection antibodies. For the orientation and the immobilization control on the microarray, a Cy5-labeled antibody (ab6563, Abcam plc., UK) was used.

#### Buffers

For the preparation of the washing and incubation buffers, bovine serum albumin (BSA) (Albumin Fraction V, biotin free, Carl RothGmbH + Co. KG, Germany), Tween 20 (for molecular biology, Sigma Aldrich, Germany) and phosphate buffered saline (PBS) (Phosphate buffered saline tablets, Sigma Aldrich, Germany) were used. The washing buffer (pH 7.4) consists of 0.1% (v/v) Tween 20 in 10 mM PBS and the incubation buffer (pH 7.4) consists of 0.1% (w/v) BSA in 10 mM PBS as albumins are still present and the main component in serum which acts as sample material.

#### Samples

Three different types of samples were used. A biomarker spiked buffer matrix, biomarker spiked human serum and real clinical patient samples. The biomarkers were spiked into the incubation buffer as well as in biomarker-free undiluted human serum. Both spiked sample types provide the identical concentrations of biomarkers. The stock solution of these samples contain 1000 pg/ml of hIL-4, hIL-6, hIL-10, hTNF-α, hIFN-γ and 2000 ng/ml of hPCT. For the detection of hIL-4, hIL-6, hIL-10, hTNF-α and hIFN-γ, the samples were 3-fold serial diluted 5 times, and for detection of hPCT the samples were additionally 10-fold serial diluted 5 times as the detectable concentration range differs for hPCT compared to the other biomarkers.

For the experiments with real patient samples, human serum samples from patients with SIRS, samples from patients with sepsis and also samples of healthy patients were used. The samples were collected and obtained by the Emergency Medicine Department, Medical Center–University of Freiburg, Germany.

### Synthesis of the copolymer

**Fig 1:**
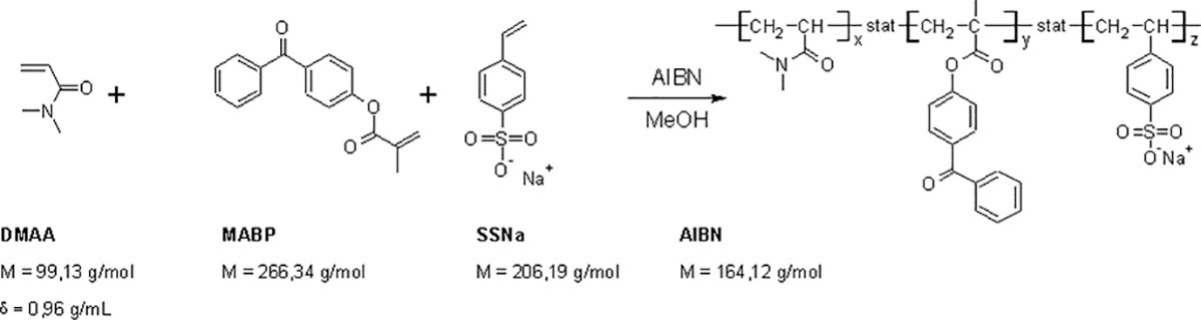
Synthesis of used copolymers P(DMAA-5%MABP-2.5%SSNa), M ≈ 300,000 g/mol [18].

The copolymer for the subsequent hydrogel based protein microarray biochip fabrication was synthesized applying a free radical polymerization as previously described [17]. In brief, the monomers N,N-dimethylacrylamide (DMAA, 92.5 mol %), methacryloyloxybenzophenone (MABP, 5 mol %) and Na -4- styrenesulfonate (SSNa, 2.5 mol %) were dissolved under nitrogen in methanol (2 M) and 0.1 mol % of α,α´-azoisobutyronitrile (AIBN) was subsequently added. For degassing, the copolymer was repeatedly frozen and thawed 5 times. The polymerization reaction took 20 hours at a constant temperature of 60°C. Subsequently, the resulting polymer was precipitated in diethylether, filtered and reprecipitated from methanol. The schematic synthesis steps are illustrated in Fig 1.

The copolymer was lyophilized and stored protected from light at 4°C until used for microarray fabrication.

#### Fabrication of hydrogel based protein microarray biochips

**Fig 2:**
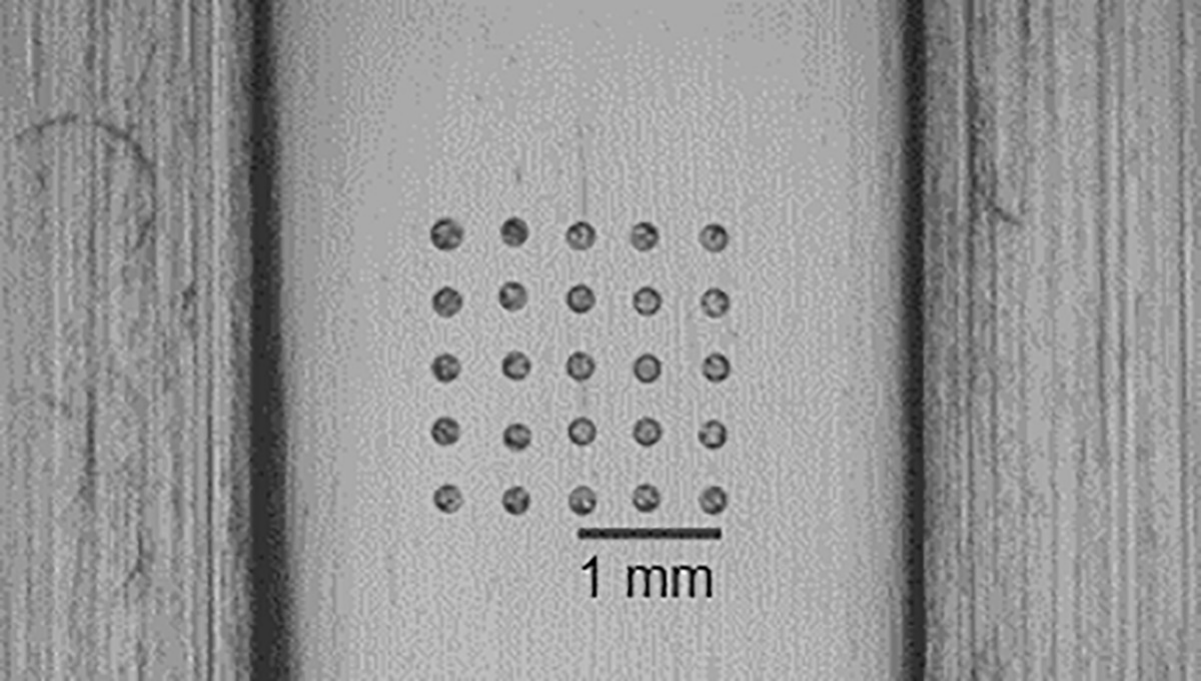
Microarray spots immobilized within microfluidic channel structure.

The microarray was fabricated applying the one-step process for immobilization of biomolecules on plastic substrates [3]. In brief, P(DMAA-5%MABP-2.5%SSNa) is used as copolymer to immobilized the capture antibodies on the commercially available unmodified COP substrates. Theoretically, all other plastic substrates, e.g. polymethylmetacrylate (PMMA), cyclo olefin copolymer (COC) or polystyrene (PS) can be used if they exhibit low background signals and are suitable for non-contact printing processes. Before the printing of the protein microarray with a sciFLEXARRAYER S3 (Scienion AG, Berlin, Germany) the print media need to be prepared. The print media are mixtures consisting of copolymer in water and the proteins in a buffer. Therefore, the lyophilised copolymer was dissolved in deionized water at a concentration of 10 mg/ml. The print media for all spots contains a final copolymer concentration of 1 mg/ml and 10 mM phosphate buffered saline (PBS, Sigma Aldrich, Germany). Fig 2 shows the Microarray after printing.

Spot 1 additionally contains 200 μg/ml Cy5-labeled IgG and acts as printing control and as “landing lights” for orientation. Spot 2 consisting of copolymer and PBS only acts as negative control. The description and composition of the additional spots are described in Table 2.

**Table 2:** Description and composition of the spots 3–20.

**Spot**	**Description**	**Capture antibody**	**Detection antibody**	**Recombinant proteins**
3–8	SIRS detection	100 μg/ml		
9–14	Control for Cy5 binding to detection antibody		5 μg/ml	
15–20	Control for protein binding to detection antibody			0.7 μg/ml

**Fig 3:**
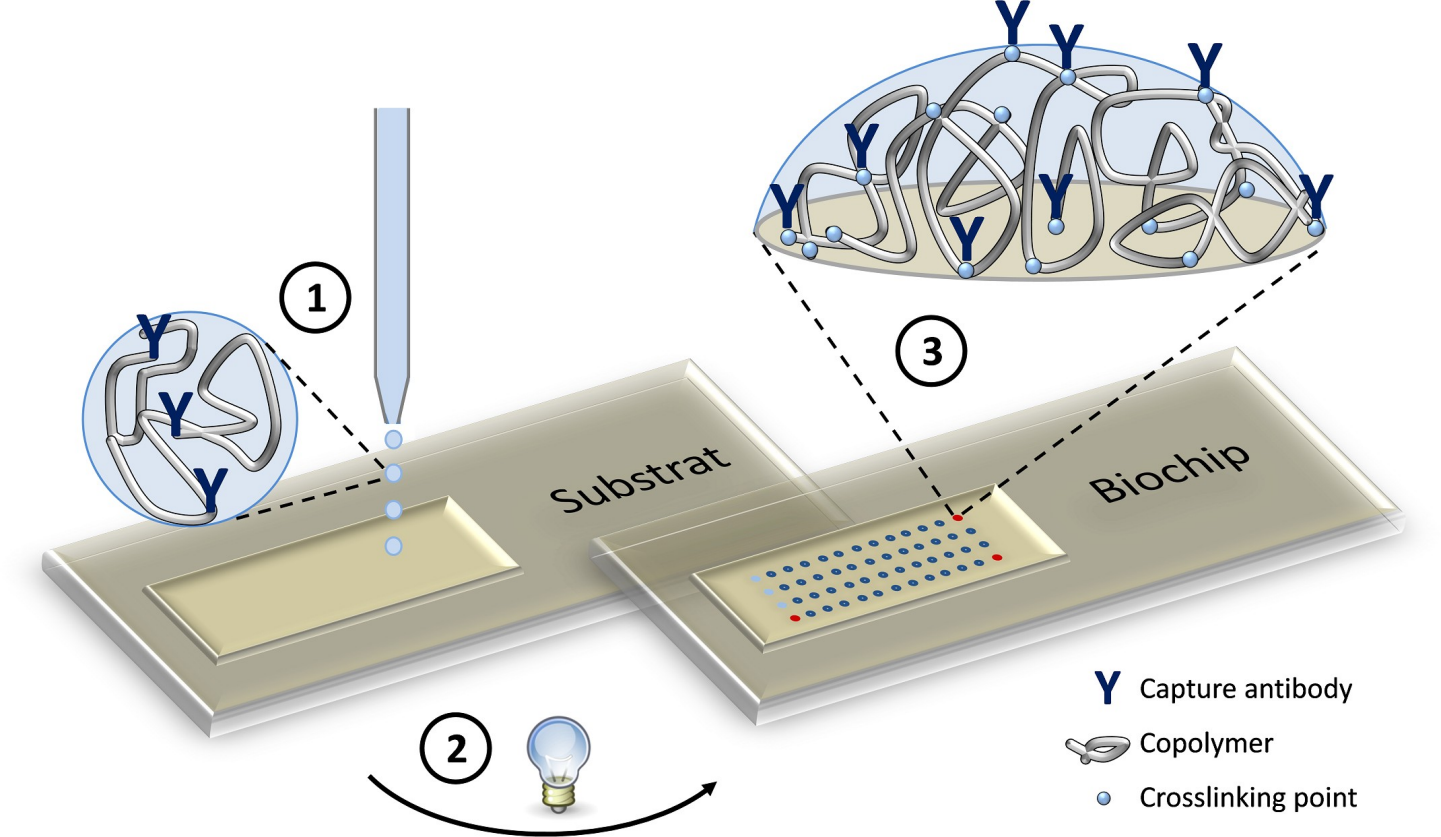
Schematic of the hydrogel based protein microarray fabrication. (1) The print media is spotted onto the unmodified COP substrate. (2) The spotted print media is irradiated with UV-light. (3) With the UV-irradiation the copolymer is crosslinked simultaneously to the COP substrate and also to the proteins in one step.

The microarray was printed into the microfluidic channel of the COP substrate and consists of 36 spots. 33 spots have a volume of 1.2 nl and a diameter of approximately 150 **μ**m per spot and because of the higher clinically relevant dynamic measurement range of hPCT the 3 hPCT spots have a volume of 2.4 nl and a diameter of approximately 250 **μ**m. At a diameter of 100–600 um, the hydrogel spots had a dry layer thickness between 20 and 100 nm, which can be regarded as thin surface-bound hydrogel layers [10]. Subsequent to the printing process, the microarray was irradiated with UV light having a wavelength of 254 nm until a dose of 1.5 Joules/cm^2^ was reached (Stratalinker, Stratagene Corp., USA, now: Agilent Technologies Inc., USA). During UV-irradiation the polymer and the proteins are covalently bonded to each other and to the substrate surface through a radical reaction process induced by the benzophenone moieties in the polymer. Thus, the process simultaneously forms the hydrogel network, binds the proteins convalently to the network and connects the forming hydrogel—as well as the therein covalently bound proteins—onto the unmodified substrate in one step. The epoxide group of the polymer reacts with the primary amino group of the proteins building a polymer-protein complex [20,21]. With the used copolymer, the hydrogel has a mesh size of 11.3 nm. Fig 3 illustrates the hydrogel based protein microarray fabrication.

After the spotting and cross-linking procedure, the microarrays were stored protected from light at 4°C until usage. Before usage, the microfluidic channel containing the protein microarray was sealed with an adhesive foil (Polyolefin foil, 900320, HJ-BIOANALYTIK GmbH, Germany) and the microarray containing channel subsequently rinsed with washing buffer (0.1% (v/v) Tween 20 in 10 mM PBS) to remove uncross-linked materials, before applying the samples.

### Immunoassay

The immunoassays were processed at room temperature. 200 μl of the washing buffer (0.1% (v/v) Tween 20 in 10 mM PBS) were used for each washing step. All the aqueous reagent solutions were transferred into the sealed microfluidic channel of the COP substrates through the inlet hole via pipetting. The calibration curves were determined with the biomarker-spiked buffer matrix samples and with the biomarker-spiked human serum samples for comparison of signal intensities and curve shapes as well as for method suitability. The sample volume was 25 μl per test. The incubation time for each sample in the microarray containing microchannel was 2 hours followed by a washing step. Subsequently, 25 μl of the biotinylated detection antibodies (2 μg/ml each) added in incubation buffer was incubated for one hour followed by 3 washing steps.

#### Labeling/Staining

For the labeling of the bound detection antibodies, a Cy5-labeled streptavidin molecule (PA45001, VWR international GmbH, Germany) was added to a 10 mM PBS buffer (final Cy5 concentration 200 μg/ml) and 25 μl were pipetted into the microarray containing COP substrate for a 5 min incubation followed by 3 washing steps. Subsequently, the sealing foil was removed and the substrates were dried with a nitrogen jet and stored at 4°C and protected from light until usage.

#### Fluorescence measurements and signal analysis

**Fig 4:**
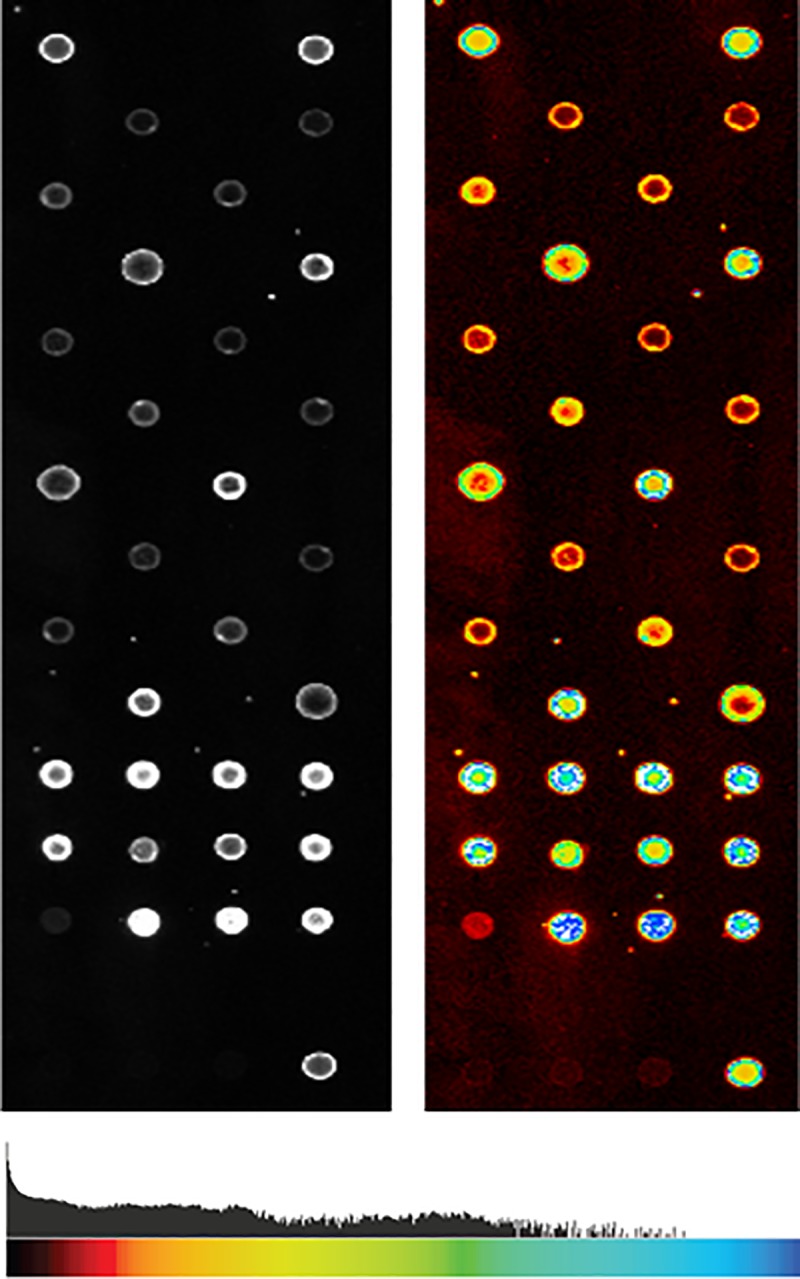
Color coding of 16-bit grayscale images in Signalyse.

The Fluorescent Array Imaging Reader (FLAIR) (Sensovation AG, Germany) with λ_Ex_ = 525–535 nm and λ_em_ = 655–665 nm and an exposure time of 5 seconds was used for fluorescent readout of the substrates (Fig 4).

In order to quantify the fluorescence signals of the microarrays, the images were evaluated by using the Software Signalyse (Klapproth und Rühe GbR, Freiburg, Germany). The gray values of all pixels of a spot point were integrated and the signal from the surrounding background was subtracted. Replicas of the same spot within a microarray were summarized as an average value and the corresponding standard deviation was determined. The background-corrected signal intensities (signal intensities minus signal intensities from a negative control) were used for data analysis. All datasets were subjected to a four-parameter logistic fit-based standard curve analysis [22] with Eq 1:

$$Y=D+\frac{A-D}{\left(1+{\left(\frac{X}{c}\right)}^{B}\right)}$$ Eq 1

where Y is the response, D is the response of the infinite analyte concentration, A is the response at zero analyte concentration, x is the analyte concentration, c is the inflection point and B is the slope factor. The fit of the curve was generated by the software OriginPro (OriginLab Corporation, USA). The limit of detection (LOD), which is defined as the lowest concentration that can be detected reliably, was calculated with the Eq 2 as recommended by the FDA guidance for bioanalytical method validation [23] [4]

LOD=c*(A−Dy0+3*σy0−D−1)1B Eq 2

where c is the value of replica at concentration, A is the background signal intensity, D is maximum signal intensity value, y_0_ is the mean value of zero concentration response, σ_y0_ is the standard deviation of zero concentration response and B is the slope factor. The LOD for each parameter of the calibration curve in buffer matrix and human serum was calculated from the mean values of three on-chip replicates and four off-chip replicates. The coefficient of variation (CV) is calculated from the standard deviation σ x 100/mean value and is the reproducibility of the assay and is also calculated for the above-mentioned replicates. The signal intensities from the patient samples were compared to the calibration curve signal in spiked human serum and the biomarker concentrations were calculated with the Eq 1.

## Results and discussion

### Immunoassay procedure with the hydrogel based protein microarray biochips

**Fig 5:**
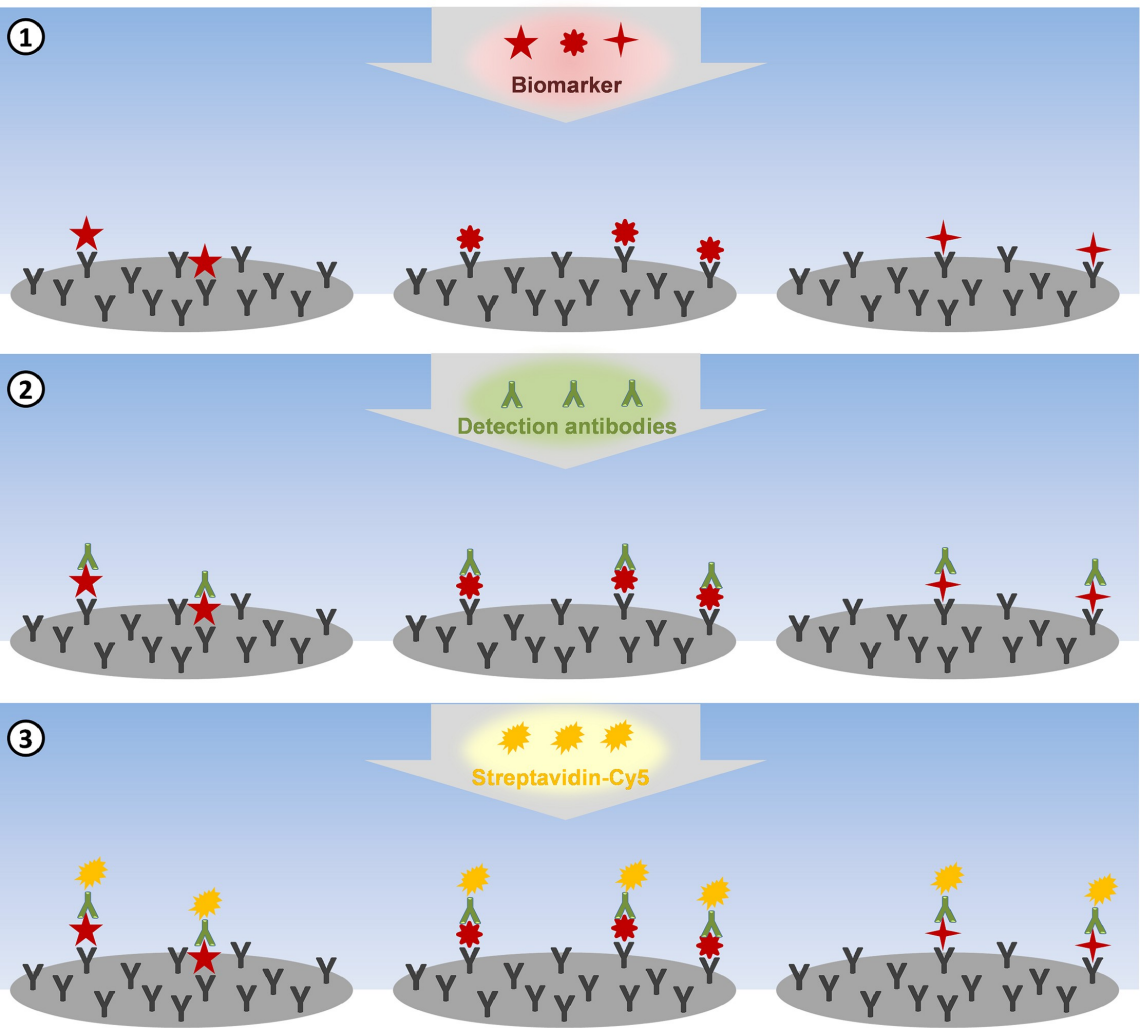
Schematic procedure of the non-competitive immunoassay. (1) The biomarker containing sample is transferred to the immobilized capture antibodies of the microarray. (2) The detection antibodies bind to the capture antibody bound biomarkers. (3) The Cy5-labeled streptavidin molecules bind to the detection antibodies, enabling the biomarker detection via fluorescence signal analysis.

In clinical diagnostics ELISA assays are the gold standard in identifying protein related biomarkers with the need of samples volumes in the ml range and the detection limit of one biomarker per ELISA test. Furthermore, the capacity of parallel biomarker detection in microtiter plates, which are generally used for ELISA, is limited by the number of wells and can only be increased by using microarrays. Immunoassays are used to detect biomarkers in liquid phase and due to the increased sensitivity we applied a non-competitive immunoassay (Fig 5) in our hydrogel based protein microarray biochips [24].

The contactless printing method allowed successful deposition of the hydrogel-based protein microarray into the microfluidic channel of the COP substrate. After the printing procedure the dispensed capture antibody-containing spots rapidly dry and the microfluidic channel was sealed with an adhesive foil prior to the non-competitive immunoassay procedure. The sealed microfluidic channel, acting as reaction chamber, has a volume of 21.6 μl. A sample volume of 25 μl was used to ensure entire filling of the reaction volume. In the first step of the immunoassay, the sample was supplied to the biochip and incubated in the microfluidic channel for 2 hours at room temperature. Subsequently, the procedure is followed by removal of non-bound molecules by a washing step, the incubation of the detection antibodies, the incubation of the Cy5-labeled streptavidin molecules and the final fluorescence signal detection.

### Analysis of spiked buffer matrix as sample

**Fig 6:**
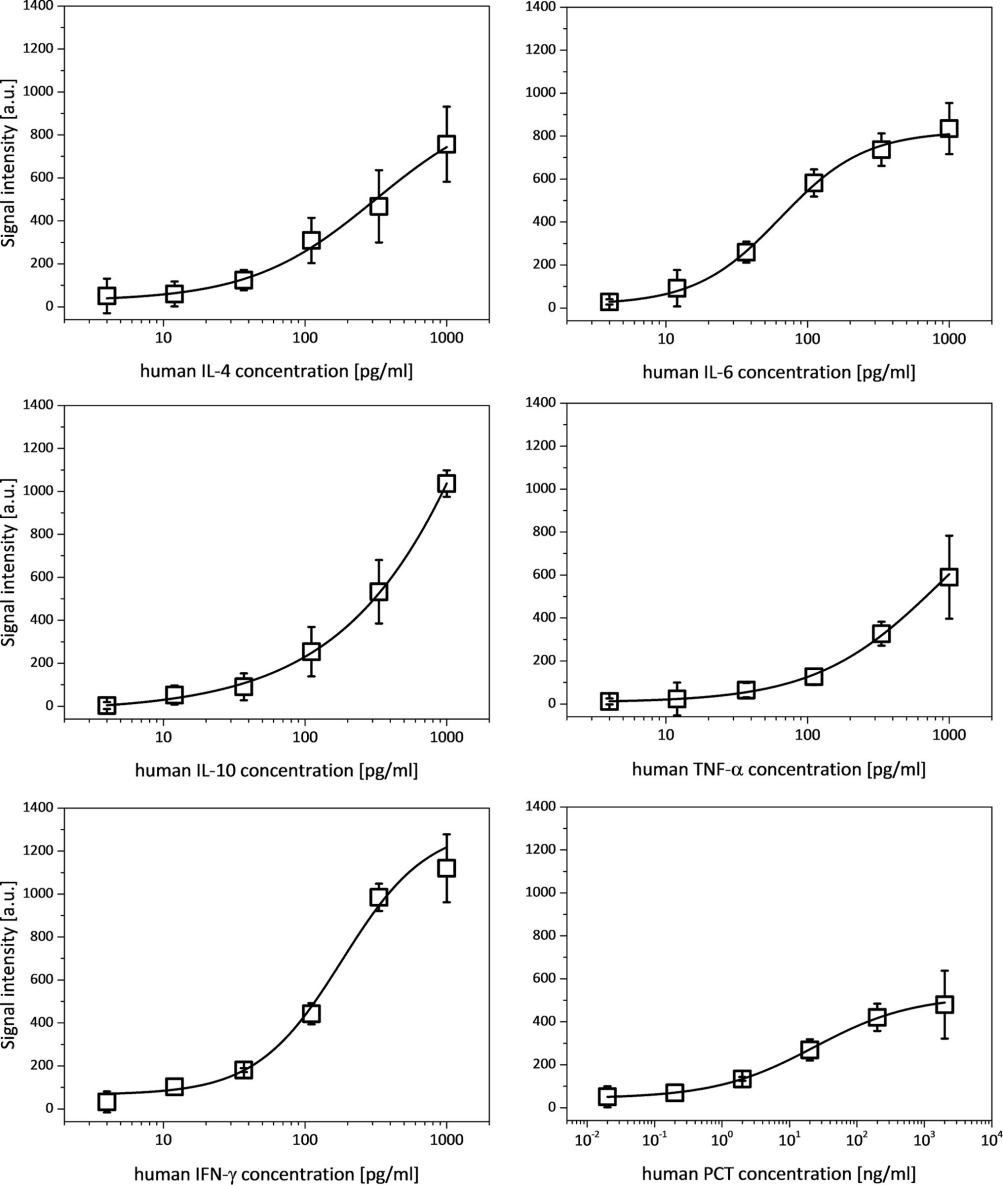
Standard curves for the six biomarkers determined for the buffer matrix samples. For hIL-4, hIL-10 and hTNF-α the saturation phase was not reached with the highest applied biomarker concentrations indicating that samples with even higher biomarker concentrations might be detectable with our hydrogel based protein microarray biochip. For hIFN-γ the standard curve starts to reach the saturation phase and for hIL-6 and hPCT the standard curves show a sigmoidal curve shape indicating that the upper LOD was reached with the highest applied concentrations of 1000 pg/ml for hIL-6 and 2000 ng/ml for hPCT.

For a SIRS diagnostic tool it is important to quantify the concentration of the relevant biomarkers in the samples to differentiate between sepsis and SIRS. Even more important is a sufficient LOD and thus a sufficient sensitivity of the test as the SIRS biomarkers are usually low abundant in sera of infected patients. Another important aspect is the simultaneous detection of the biomarkers hIL-4, hIL-6, hIL-10, hTNF-α and hIFN-γ in the clinically relevant pg/ml range and hPCT with clinically relevant concentrations from pg/ml up to the μg/ml range. There are sophisticated approaches for the simultaneous detection of biomarkers in a broad range of concentrations by combination of two different immunoassay formats, a competitive and a sandwich immunoassay [17,19,20] [5-8]. Our detection system using the hydrogel based protein microarray enables sufficient sensitivity for the successful simultaneous detection of the different clinically relevant concentrations without complex adaptions of the immunoassay procedure as the required detectable concentration range can easily be adjusted during the biochip fabrication process by varying the spot volume. Each microarray contains 3 spots of immobilized capture antibodies for each biomarker (see Fig 2) enabling parallel detection of triplicates of each biomarker per substrate. Four substrates were analyzed and thus 12 measurements for each biomarker were considered to determine the signal intensity for six different biomarker concentrations. Initially, the applicability of the test was validated with spiked buffer matrix samples before applying spiked human serum samples as well as clinical patient samples. The standard curves for the spiked buffer matrix samples are illustrated in Fig 6.

For hIL-4, hIL-10 and hTNF-α the standard curves did not reach the saturation phase with the highest applied sample concentration of 1000 pg/ml indicating that the upper LOD of the hydrogel based protein microarray could not be determined for these 3 biomarkers and thus enabling detectable concentrations which are even higher than applied here. The standard curve for hIFN-γ starts to go over into the saturation phase while the standard curves for hIL-6 and hPCT show a sigmoidal curve shape reaching the saturation phase for the highest applied concentration of 1000 pg/ml for hIFN-γ and hIL-6 and 2000 ng/ml for hPCT indicating that the upper LOD was reached.

The achieved results demonstrate successful parallel detection of all six biomarkers with the developed hydrogel based protein microarray biochip. A major benefit of the developed microarray is its flexibility as it enables dynamic detection of a broad range of biomarker concentrations, which can be easily adjusted by varying the spot volumes during the microarray fabrication. The dynamic measurement range covers concentrations from pg/ml up to the μg/ml range for hPCT and the pg/ml range for the other five biomarkers.

The measured signal intensities and Eqs 1 and 2 were used to determine the lower LOD of each biomarker for the detection system using the spiked buffer samples. The achieved lower LOD using the spiked buffer matrix samples are summarized in Table 3.

**Table 3:** The achieved lower LOD using spiked buffer matrixes as sample on our hydrogel based protein microarray biochip demonstrate sufficient sensitivity for clinical relevance. The lower LOD demonstrate lower detectable biomarker concentrations than the mean biomarker concentration in samples of SIRS patients.

Biomarker	Achieved lower LOD using spiked buffer samples (n = 12)	Biomarker concentrations in samples of SIRS patients given as mean ± STD
hIL-4	16.2 pg/ml	144.8 pg/ml; n = 102 [25]
hIL-6	29.5 pg/ml	61.4 pg/ml; n = 102 [25]
hIL-10	15.5 pg/ml	136.4 **±** 260.7 pg/ml; n = 33 [25]
hTNF-α	89.6 pg/ml	144.3 **±** 148.3 pg/ml; n = 43 [26]
hIFN-γ	43.3 pg/ml	74.0 pg/ml n = 102 [26]
hPCT	0.9 ng/ml	1.3 **±** 0.2 ng/ml; n = 100 [27]

Furthermore, Table 3 shows biomarker concentrations of SIRS patient samples which were collected in previous studies [25–27] indicating the clinically relevant ranges.

The determined lower LOD of the hydrogel-based protein microarray biochip for the 6 biomarkers hIL-4, hIL-6, hIL10, hTNF-α, hIFN-γ and hPCT using spiked buffer samples demonstrated sufficient sensitivity for clinical usage showing lower values as the mean biomarker concentrations in samples of patients suffering on SIRS.

### Analysis with undiluted human serum spiked with biomarkers as sample

The characterization of the developed system with spiked human serum samples is of major interest since human serum is the matrix used for the diagnostic of SIRS. As the SIRS-related biomarkers are low abundant proteins, a highly sensitive detection platform is required for human serum sample and furthermore, it is essential to use undiluted human serum in order to demonstrate detection of clinically relevant samples. To achieve a clinically relevant detection range and lower LOD, the assay has to provide a detection platform with sufficient sensitivity. The most difficult hurdle which will be necessary to overcome for the successful usage of human serum samples is the influence of the interaction of the serum-contained proteins with the biomarkers and the detection platform [27]. The interaction with the detection platform might result in increased background signals and thus in a reduced sensitivity of the detection. Furthermore, the interaction of the human serum proteins with the biomarkers could results in a lower amount of bound biomarkers and thus in lower signal intensities which might lead to a higher LOD values. To circumvent these problems which occurred through the interaction of the serum proteins with the biomarkers and the detection platform, other already published studies [28,29] used diluted serum samples resulting in a comparatively low sensitivity which usually does not result in clinically relevant LOD. With our newly developed detection platform, undiluted human serum samples can be tested so that a sufficient sensitivity for the detection of low abundant biomarker is achieved. This is mandatory for the SIRS related biomarker detection using clinical patient samples. To verify the LOD and the sensitivity of the hydrogel based protein microarray biochip, calibration curves of all biomarkers were generated for the determination of the LOD and the CV values with spiked undiluted human serum samples. The determination of the standard curves with the spiked human serum are required for the concentration determination of the subsequently tested clinical patient samples.

**Fig 7:**
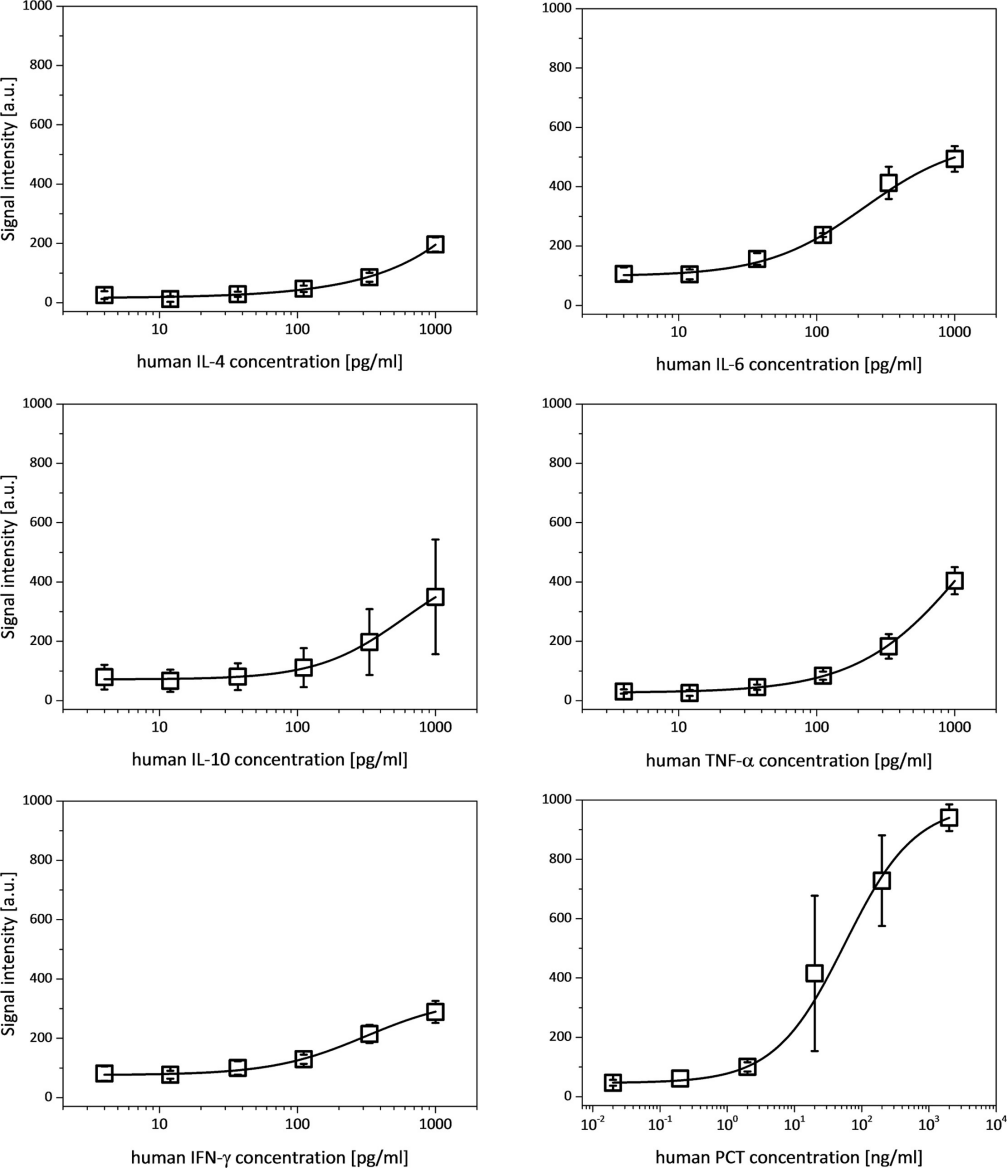
Standard curves for the six biomarkers determined for the undiluted human serum samples spiked with biomarkers. For hIL-4, hIL-10 and hTNF-α the saturation phase was not reached with the highest applied sample concentrations of 1000 pg/ml. Therefore, even higher biomarker concentrations in human serum samples might be detectable with our hydrogel based protein microarray biochip. For hIL-6, hIFN-γ and hPCT the standard curve starts to show a sigmoidal curve shape and begin to reach the saturation phase, indicating that the highest applied concentrations of 1000 pg/ml for hIL-6 and hIFN-γ and 2000 ng/ml for hPCT are close to the upper LOD.

Four substrates were analyzed with undiluted human serum samples and thus 12 measurements for each biomarker were used to determine the signal intensity for six different biomarker concentrations. The standard curves for the spiked human serum samples are shown in Fig 7.

The detected signal intensities of the biomarkers in spiked human serum (Fig 7) are—as expected—lower compared to the signal intensities measured with spiked buffer samples, indicating a reduced amount of bound molecules [30]. However, the signal intensities are sufficient for successful detection of all six biomarkers. The standard curves of hIL-4, hIL-10 and hTNF-α did not reach the saturation phase with the highest applied sample concentration of 1000 pg/ml indicating that the upper LOD of the hydrogel based protein microarray could not be determined for these 3 biomarkers and thus even higher biomarker concentrations in human serum might be detectable. The standard curves for hIL-6, hIFN-γ and hPCT are at the beginning of the saturation phase for the highest applied concentration of 1000 pg/ml for hIL-6 and hIFN-γ and 2000 ng/ml for hPCT indicating that these biomarker concentrations are close to the upper LOD. The results demonstrate successful parallel detection of all six biomarkers with the developed hydrogel based protein microarray biochip using spiked human serum samples and the dynamic measurement range covers detectable concentrations from pg/ml up to the μg/ml range for hPCT and the pg/ml range for hIL-4, hIL-6, hIL-10, hTNF-α and hIFN-γ. The lower LOD of each biomarker for the detection system using the spiked human serum samples were determined and are stated in Table 4.

**Table 4:** The achieved lower LOD using spiked human serum as sample on our hydrogel based protein microarray biochip demonstrate sufficient sensitivity for clinical relevance. For all 6 biomarkers lower LOD values as the mean biomarker concentrations in samples of patients suffering on SIRS was determined.

Biomarker	Achieved lower LOD using spiked human serum samples (n = 12)	Biomarker concentrations in samples of SIRS patients given as mean ± STD
**hIL-4**	75.2 pg/ml	144.8 pg/ml; n = 102 [25]
**hIL-6**	45.1 pg/ml	61.4 pg/ml; n = 102 [25]
**hIL-10**	71.5 pg/ml	136.4 **±** 260.7 pg/ml; n = 33 [25]
**hTNF-α**	56.7 pg/ml	144.3 **±** 148.3 pg/ml; n = 43 [26]
**hIFN-γ**	46.4 pg/ml	74.0 pg/ml; n = 102 [26]
**hPCT**	1.1 ng/ml	1.3 **±** 0.2 ng/ml; n = 100 [27]

Additionally, Table 4 shows biomarker concentrations of SIRS patient samples which were determined in previous studies [25–27] and indicating the range of clinical relevance.

The determined lower LOD of the hydrogel-based protein microarray biochip for the 5 biomarkers hIL-4, hIL-6, hIL10, hIFN-γ and hPCT using spiked human serum samples are higher than the lower LOD of the spiked buffer samples. However, the increased LOD for the undiluted human serum samples spiked with biomarkers was expected and have no negative impacts as all determined signal intensities and lower LOD values are still demonstrating sufficient sensitivity for clinical relevance. For all 6 biomarkers lower LOD values as the mean biomarker concentrations in samples of patients suffering on SIRS was determined.

### Analysis with clinical patient samples

Regarding the proof of concept for real world applicability of the developed hydrogel based protein microarray biochip, it is essential to apply the immunoassay for biomarker detection under real conditions with undiluted clinical patient samples. Therefore, the developed hydrogel based protein microarray biochip was tested with undiluted serum samples from healthy persons as well as from SIRS patients. To compare the results of the hydrogel based protein microarray biochip with results determined by the “gold standard” ELISA reference method, all clinical patient samples were additionally analyzed by ELISA.

**Fig 8:**
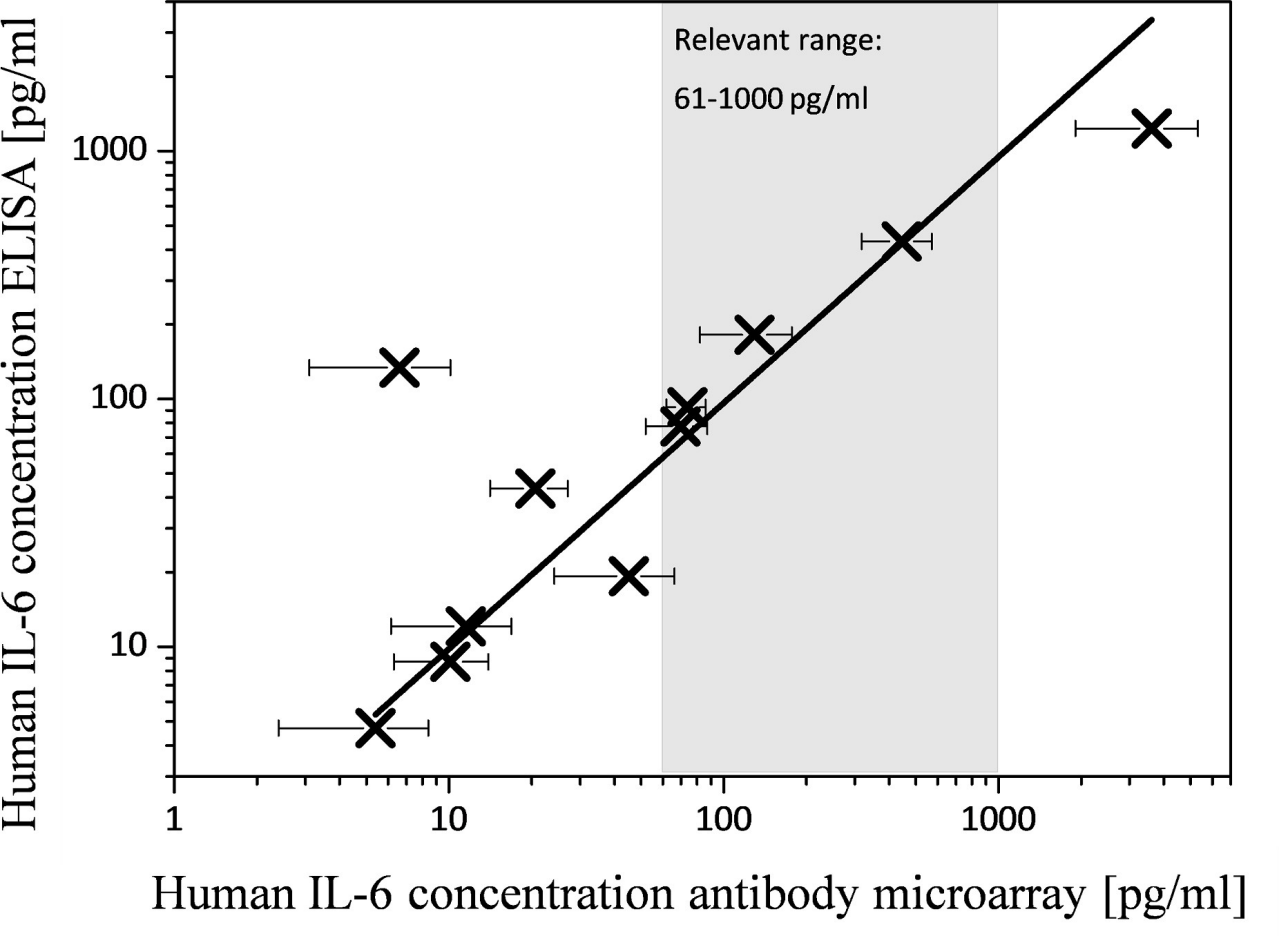
Comparison of the measured hIL-6 concentrations of the reference ELISA and the hydrogel based protein microarray biochip. In total 11 different clinical patient samples were analyzed.

In total, 11 individual clinical patient samples were used and each samples was tested with two hydrogel-based protein microarray biochips resulting in a total of 22 tests. For each clinical patient sample the mean value and standard deviation was determined by measuring the 6 signal intensities per biomarker (2 substrates per sample, 3 measured biomarker replicates per substrate). As the reference ELISA measurement requires a high sample volume of 3 ml for the measurement, only two biomarkers, hIL-6 and hPCT, were chosen for proof of concept with our hydrogel based protein microarray. These two biomarkers enabling the demonstration of parallel biomarker detection with the biochip in a wide range of concentrations as hIL-6 concentrations values in the pg/ml range und hPCT concentrations values from pg/ml up to the ng/ml range. Considering the required sample volume, one ELISA was applied for each clinical patient sample. The ELISA has been carried out with the standardized procedure by the University Freiburg Medical Center clinical laboratory. Subsequently, the samples were numbered with increasing biomarker concentration (sample 1 provides lowest biomarker concentration according to ELISA result), frozen at -20°C and provided to us for our biochip tests. The comparison of the measured hIL-6 concentrations of the biochip and the reference ELISA are illustrated in Fig 8.

For hIL-6 one sample shows a 20 times lower concentration compared to the ELISA result. The date were fitted using a linear regression through the origin and the determined hIL-6 corrected correlation coefficient R^2^ [31] [9] for the comparison of our hydrogel based protein microarray biochip with the reference ELISA is 0.94.

**Fig 9:**
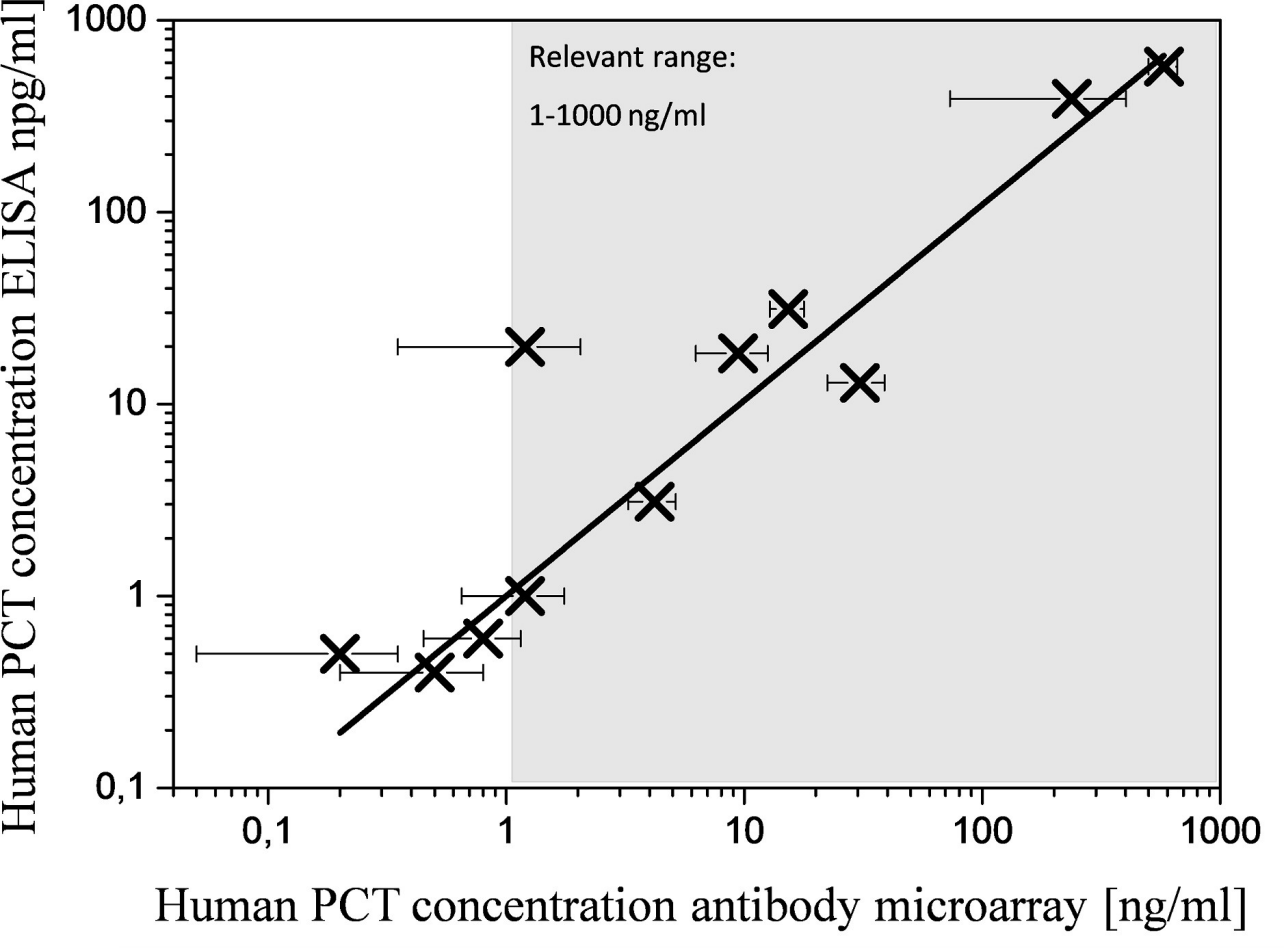
Comparison of the measured hPCT concentrations of the reference ELISA and the hydrogel based protein microarray biochip. In total 11 different clinical patient samples were analyzed.

The comparison of the measured hPCT concentrations of the biochip and the reference ELISA are illustrated in Fig 9.

For hPCT the biomarker concentrations of one sample is approximately 17 times decreased compared to the ELISA determined concentration. The determined data are interpolated with a straight line through the origin (y = a+b*x, a = 0) and for the biomarker hPCT the corrected correlation coefficient R^2^ for the tests with clinical patient samples is 0.78. This indicates a sufficient correlation between the ELISA and the hydrogel protein microarray based biochip [32].

The divergences in the biomarker concentrations identified might be caused by negatively influencing interference reactions of the proteins of the serum and the patient sample biomarkers [27] and could also generally be attributed to the nature of proteins and their independent behavior. For sample 9, affecting hIL-6 and hPCT it is assumed that the divergences are not related to the detection method but rather related to the sample material or rather the sample matrix. Regarding immunoassays, studies found that varying or misleading measurements are related to interferences of the matrix with the biomarkers [33] attributed to a matrix effect whose exact molecular mechanism is not known. Possible reasons for the interferences might be the salt concentrations, pH values or the viscosity which all are different from sample to sample [34]. However, the hIL-6 and hPCT concentrations which were determined by the hydrogel-based protein microarray biochips shows an overall accordance with the ELISA determined concentrations. Furthermore, the detected biomarker concentrations were in the range of clinical relevance. The achieved R^2^ values demonstrated a linear relation between this two detection methods and accordance and correlation of our hydrogel based protein microarray with the “gold standard” ELISA. The successful parallel detection of hIL-6 and hPCT by our hydrogel based protein microarray biochip using clinical patient human serum samples was demonstrated and showed accordance with the “gold standard” ELISA. The detected concentrations were in the range of clinical relevance demonstrating applicability of our protein microarray biochip for SIRS related biomarker detection in patient samples in less than 200 minutes.

## Conclusions

We developed a hydrogel based protein microarray which is spotted on an unmodified COP substrate with an easy, fast and cost-efficient one-step immobilization procedure of copolymer and biomarkers. The microarray allows parallel detection of six biomarkers used for the diagnosis of SIRS. Successful parallel detection of the low abundant biomarkers hIL-4, hIL-6, hIL-10, hTNF-α and hIFN-γ and the medium abundant hPCT with different sample materials were demonstrated. The six biomarkers were detected in spiked buffer solution as well as in spiked human serum with a LOD in the range of clinical relevance. Clinical patient samples were used for proof-of-concept experiments demonstrating parallel detection of hIL-6 and hPCT with our hydrogel-based protein microarray biochip. The results of the protein microarray biochip with the clinical patient samples were compared with the “gold standard” method ELISA used as a reference measurement for these samples. For the biomarker hIL-6 the corrected correlation coefficient R^2^ resulted in 0.94 and for the biomarker hPCT the corrected correlation coefficient R^2^ resulted in 0.78. A drawback of the “gold standard” ELISA method is the high sample volume of around 3 ml required for testing all six biomarkers whereas the required sample volume for the parallel biomarker detection with the developed hydrogel based protein microarray biochip is 25 μl. With our microarray detection system the parallel sample-to-answer detection of the six biomarkers is done in less than 200 minutes. This is a major benefit for the patients as a fast diagnosis of SIRS leads to a fast and targeted treatment of the disease. No sample dilution is required enabling detection of low abundant biomarkers with an LOD in the clinical range.

In future works a microfluidic automation could be developed for automated sample processing with washing and incubation procedures reducing the manual hands-on time. As this might also increase accuracies of applied volumes, the automated procedure can further reduce the CV values and will allow applicability as an easy-to use point-of-care system for SIRS diagnosis. Furthermore, additional biomarkers could be added for a better detection of SIRS, sepsis as well as other diseases with the hydrogel based protein microarray biochip.
